# Supplementary material for: Comparative Analysis of Compound Probiotics, Seasonal Variation, and Age on Gut Microbial Composition and Function in Endangered Forest Musk Deer
Source: Microorganisms. 2025 Aug 26;13(9):1991. doi: 10.3390/microorganisms13091991 (PMC12471530; doi:10.3390/microorganisms13091991)

Table S1 Composition proportions of gut microbiota in forest musk deer under different influencing factors at the phylum level. G1 and G2 represented the adult and juvenile groups fed with composite probiotics during the summer, while G3 and G4 represented the adult and juvenile groups not fed with composite probiotics during the summer. G5 and G6 indicated the adult and juvenile groups not fed with composite probiotics during the winter. Phyla with a relative abundance greater than 1% were considered dominant and were indicated in bold.

	G1 group	G2 group	G3 group	G4 group	G5 group	G6 group
Firmicutes/%	**59.10**	**35.17**	**66.42**	**71.20**	**81.92**	**80.15**
Bacteroidota/%	**19.75**	**15.22**	**29.02**	**25.23**	**14.93**	**17.19**
Proteobacteria/%	**15.13**	**45.38**	**2.32**	0.77	0.36	0.25
Actinobacteriota/%	**4.41**	**3.18**	0.84	**1.13**	**1.15**	0.99
Planctomycetota/%	**1.08**	0.44	0.34	0.79	0.99	0.53
Spirochaetota/%	0.07	0.09	0.09	0.16	0.05	0.04
Verrucomicrobiota/%	0.05	0.03	0.15	0.08	0.08	0.09
Campilobacterota/%	0.00	0.00	0.00	0.00	0.03	0.06
Others/%	0.40	0.49	0.80	0.64	0.50	0.69

Table S2 Composition proportions of the top 20 identifiable bacterial genera in the gut microbiota of forest musk deer under different influencing factors. Genera with a relative abundance greater than 1% were considered dominant and were indicated in bold.

	G1 group	G2 group	G3 group	G4 group	G5 group	G6 group
Christensenellaceae R-7 group/%	**10.26**	**4.00**	**8.67**	**9.17**	**18.22**	**17.69**
UCG-005/%	**11.46**	**7.56**	**14.48**	**11.64**	**9.23**	**10.5**
Acinetobacter/%	**10.93**	**31.22**	**2.07**	0.29	0.00	0.00
Bacteroides/%	**8.15**	**4.54**	**9.48**	**6.65**	**2.75**	**3.49**
Rikenellaceae RC9 gut group/%	**2.32**	0.93	**4.36**	**2.56**	**5.03**	**6.02**
Alistipes/%	**3.44**	**1.63**	**3.73**	**2.45**	**2.71**	**2.59**
NK4A214 group/%	**3.7**	**2.7**	**1.89**	**2.19**	**1.5**	**1.44**
Prevotellaceae UCG-004/%	**1.06**	0.48	**2.72**	**2.59**	**2.48**	**2.19**
Ruminococcus/%	**1.51**	0.59	**1.78**	**1.66**	**2.53**	**2.06**
Monoglobus/%	**1.46**	**1.08**	**1.8**	**3.01**	**1.33**	**1.38**
Lachnospiraceae AC2044 group/%	0.31	0.15	0.52	0.83	**1.59**	**1.11**
Escherichia-Shigella/%	0.91	**3.21**	0.01	0.01	0.00	0.00
p-1088-a5 gut group/%	**1.07**	0.42	0.34	0.78	0.97	0.48
dgA-11 gut group/%	**1.06**	0.5	**1.03**	0.79	0.37	0.22
Arthrobacter/%	**3.06**	**1.38**	0.04	0.26	0.01	0.00
Solibacillus/%	**1.56**	**2.26**	0.07	0.02	0.00	0.00
UCG-002/%	0.46	0.21	0.58	0.43	0.57	**1.06**
Comamonas/%	0.29	**2.69**	0.01	0.02	0.00	0.00
Flavobacterium/%	0.07	**2.41**	0.02	0.12	0.00	0.00
Sphingobacterium/%	0.02	**1.45**	0.01	0.22	0.00	0.00

Table S3 Analysis of shared and unique OTU counts and proportions across different groups under various influencing factors.

Comparison Type	Groups	Group1	Shared	Group2	Total	Shared Proportion	Unique Proportion
Probiotic groups	G1-G3	141	2251	408	2800	80.39 %	19.61 %
Probiotic groups	G2-G4	202	2300	352	2854	80.59 %	19.41 %
Seasonal groups	G3-G5	238	2421	110	2769	87.43 %	12.57 %
Seasonal groups	G4-G6	212	2440	109	2761	88.37 %	11.63 %
Age groups	G1-G2	170	2222	280	2672	83.16 %	16.84 %
Age groups	G3-G4	130	2529	123	2782	90.91 %	9.09 %
Age groups	G5-G6	95	2436	113	2644	92.13 %	7.87 %

Table S4 Intergroup difference analysis between G1 and G2 using ANOSIM and Adonis test based on 29 different distance algorithms.

Distance algorithms	ANOSIM test		Adonis test	
	R value	*P*	R^2 value	*P*
Bray curtis	0.381	0.002	0.155	0.001
Unweighted unifrac	0.087	0.136	0.094	0.001
Weighted unifrac	0.340	0.002	0.269	0.001
Euclidean	0.274	0.006	0.165	0.005
Abund jaccard	0.274	0.003	0.302	0.002
Bray curtis faith	0.381	0.002	0.155	0.001
Bray curtis magurran	0.381	0.002	0.155	0.001
Canberra	0.049	0.245	0.058	0.001
Chisq	0.170	0.048	0.071	0.001
Chord	0.501	0.001	0.189	0.001
Hellinger	0.366	0.002	0.134	0.001
Kulcynski	0.381	0.002	0.155	0.001
Manhattan	0.381	0.002	0.155	0.001
Morista horn	0.552	0.001	0.289	0.001
Person	0.501	0.001	0.310	0.001
Soergel	0.381	0.002	0.104	0.001
Spearman approx	0.401	0.002	0.123	0.001
Specprof	0.274	0.006	0.165	0.005
Unweighted unifrac full tree	0.258	0.006	0.123	0.001
Weighted normalized unifrac	0.432	0.001	0.276	0.001
Binary serensen dice	0.039	0.279	0.105	0.001
Binary chisq	-0.031	0.601	0.057	0.004
Binary chord	0.030	0.311	0.069	0.001
Binary euclidean	0.463	0.001	0.078	0.001
Binary hamming	0.463	0.001	0.123	0.001
Binary jaccard	0.039	0.279	0.084	0.001
Binary lennon	-0.006	0.471	0.066	0.029
Binary ochiai	0.030	0.311	0.101	0.001
Binary pearson	0.247	0.005	0.104	0.001

Table S5 Intergroup difference analysis between G3 and G4 using ANOSIM and Adonis test based on 29 different distance algorithms

Distance algorithms	ANOSIM test		Adonis test	
	R value	*P*	R^2 value	*P*
Bray curtis	0.080	0.008	0.039	0.008
Unweighted unifrac	0.034	0.134	0.031	0.129
Weighted unifrac	0.049	0.051	0.049	0.032
Euclidean	0.045	0.026	0.037	0.072
Abund jaccard	0.050	0.038	0.042	0.106
Bray curtis faith	0.080	0.008	0.039	0.008
Bray curtis magurran	0.080	0.008	0.039	0.008
Canberra	0.087	0.004	0.031	0.002
Chisq	0.020	0.075	0.033	0.007
Chord	0.018	0.224	0.031	0.183
Hellinger	0.070	0.013	0.038	0.008
Kulcynski	0.080	0.008	0.039	0.008
Manhattan	0.080	0.008	0.039	0.008
Morista horn	0.034	0.113	0.040	0.089
Person	0.017	0.241	0.033	0.207
Soergel	0.080	0.008	0.035	0.008
Spearman approx	0.067	0.017	0.037	0.018
Specprof	0.045	0.026	0.037	0.072
Unweighted unifrac full tree	0.050	0.084	0.032	0.142
Weighted normalized unifrac	0.042	0.072	0.048	0.036
Binary serensen dice	0.065	0.011	0.036	0.011
Binary chisq	0.036	0.021	0.030	0.014
Binary chord	0.065	0.011	0.031	0.008
Binary euclidean	0.085	0.007	0.031	0.008
Binary hamming	0.085	0.007	0.036	0.011
Binary jaccard	0.065	0.011	0.034	0.008
Binary lennon	0.050	0.040	0.034	0.038
Binary ochiai	0.065	0.011	0.036	0.010
Binary pearson	0.082	0.008	0.036	0.012

Table S6 Intergroup difference analysis between G5 and G6 using ANOSIM and Adonis test based on 29 different distance algorithms

Distance algorithms	ANOSIM test		Adonis test	
	R value	*P*	R^2 value	*P*
Bray curtis	0.031	0.108	0.035	0.021
Unweighted unifrac	0.170	0.006	0.056	0.002
Weighted unifrac	-0.030	0.854	0.021	0.478
Euclidean	-0.010	0.687	0.022	0.643
Abund jaccard	0.075	0.006	0.074	0.002
Bray curtis faith	0.031	0.108	0.035	0.021
Bray curtis magurran	0.031	0.108	0.035	0.021
Canberra	0.091	0.005	0.032	0.002
Chisq	0.011	0.208	0.031	0.011
Chord	-0.007	0.547	0.025	0.471
Hellinger	0.043	0.058	0.036	0.008
Kulcynski	0.031	0.108	0.035	0.021
Manhattan	0.031	0.108	0.035	0.021
Morista horn	-0.013	0.649	0.024	0.526
Person	-0.006	0.539	0.025	0.477
Soergel	0.031	0.108	0.032	0.023
Spearman approx	0.158	0.001	0.053	0.001
Specprof	-0.010	0.687	0.022	0.643
Unweighted unifrac full tree	0.201	0.006	0.063	0.002
Weighted normalized unifrac	-0.032	0.861	0.021	0.524
Binary serensen dice	0.102	0.001	0.045	0.001
Binary chisq	0.077	0.001	0.034	0.001
Binary chord	0.104	0.001	0.035	0.001
Binary euclidean	0.141	0.001	0.035	0.001
Binary hamming	0.141	0.001	0.044	0.001
Binary jaccard	0.102	0.001	0.040	0.001
Binary lennon	0.155	0.001	0.051	0.001
Binary ochiai	0.104	0.001	0.045	0.001
Binary pearson	0.138	0.001	0.045	0.001

Table S7 Intergroup difference analysis between G3 and G5 using ANOSIM and Adonis test based on 29 different distance algorithms

Distance algorithms	ANOSIM test		Adonis test	
	R value	*P*	R^2 value	*P*
Bray curtis	0.620	0.001	0.142	0.001
Unweighted unifrac	0.362	0.001	0.088	0.001
Weighted unifrac	0.457	0.001	0.267	0.001
Euclidean	0.261	0.001	0.107	0.001
Abund jaccard	0.351	0.001	0.140	0.001
Bray curtis faith	0.620	0.001	0.142	0.001
Bray curtis magurran	0.620	0.001	0.142	0.001
Canberra	0.601	0.001	0.066	0.001
Chisq	0.229	0.001	0.073	0.001
Chord	0.405	0.001	0.107	0.001
Hellinger	0.575	0.001	0.124	0.001
Kulcynski	0.620	0.001	0.142	0.001
Manhattan	0.620	0.001	0.142	0.001
Morista horn	0.432	0.001	0.187	0.001
Person	0.407	0.001	0.176	0.001
Soergel	0.620	0.001	0.100	0.001
Spearman approx	0.599	0.001	0.141	0.001
Specprof	0.261	0.001	0.107	0.001
Unweighted unifrac full tree	0.389	0.001	0.106	0.001
Weighted normalized unifrac	0.473	0.001	0.279	0.001
Binary serensen dice	0.486	0.001	0.110	0.001
Binary chisq	0.280	0.001	0.057	0.001
Binary chord	0.485	0.001	0.068	0.001
Binary euclidean	0.598	0.001	0.070	0.001
Binary hamming	0.598	0.001	0.114	0.001
Binary jaccard	0.486	0.001	0.089	0.001
Binary lennon	0.347	0.001	0.090	0.001
Binary ochiai	0.485	0.001	0.108	0.001
Binary pearson	0.570	0.001	0.110	0.001

Table S8 Intergroup difference analysis between G4and G6 using ANOSIM and Adonis test based on 29 different distance algorithms

Distance algorithms	ANOSIM test		Adonis test	
	R value	*P*	R^2 value	*P*
Bray curtis	0.452	0.001	0.110	0.001
Unweighted unifrac	0.322	0.001	0.087	0.001
Weighted unifrac	0.377	0.001	0.185	0.001
Euclidean	0.160	0.001	0.080	0.001
Abund jaccard	0.201	0.001	0.102	0.001
Bray curtis faith	0.452	0.001	0.110	0.001
Bray curtis magurran	0.452	0.001	0.110	0.001
Canberra	0.464	0.001	0.056	0.001
Chisq	0.130	0.001	0.060	0.001
Chord	0.311	0.001	0.090	0.001
Hellinger	0.431	0.001	0.101	0.001
Kulcynski	0.452	0.001	0.110	0.001
Manhattan	0.452	0.001	0.110	0.001
Morista horn	0.303	0.001	0.136	0.001
Person	0.315	0.001	0.141	0.001
Soergel	0.452	0.001	0.081	0.001
Spearman approx	0.498	0.001	0.112	0.001
Specprof	0.160	0.001	0.081	0.001
Unweighted unifrac full tree	0.407	0.001	0.105	0.001
Weighted normalized unifrac	0.381	0.001	0.189	0.001
Binary serensen dice	0.347	0.001	0.086	0.001
Binary chisq	0.192	0.001	0.049	0.001
Binary chord	0.351	0.001	0.056	0.001
Binary euclidean	0.468	0.001	0.056	0.001
Binary hamming	0.468	0.001	0.086	0.001
Binary jaccard	0.347	0.001	0.071	0.001
Binary lennon	0.351	0.001	0.091	0.001
Binary ochiai	0.351	0.001	0.086	0.001
Binary pearson	0.454	0.001	0.086	0.001

Table S9 Intergroup difference analysis between G1 and G3 using ANOSIM and Adonis test based on 29 different distance algorithms

Distance algorithms	ANOSIM test		Adonis test	
	R value	*P*	R^2 value	*P*
Bray curtis	0.369	0.001	0.114	0.001
Unweighted unifrac	0.260	0.005	0.088	0.001
Weighted unifrac	0.329	0.003	0.169	0.001
Euclidean	0.182	0.052	0.122	0.001
Abund jaccard	0.334	0.001	0.328	0.001
Bray curtis faith	0.369	0.001	0.114	0.001
Bray curtis magurran	0.369	0.001	0.114	0.001
Canberra	0.370	0.001	0.059	0.001
Chisq	0.233	0.023	0.075	0.001
Chord	0.327	0.002	0.110	0.001
Hellinger	0.392	0.001	0.112	0.001
Kulcynski	0.369	0.001	0.114	0.001
Manhattan	0.369	0.001	0.114	0.001
Morista horn	0.320	0.002	0.149	0.001
Person	0.329	0.002	0.168	0.001
Soergel	0.369	0.001	0.083	0.001
Spearman approx	0.430	0.001	0.116	0.001
Specprof	0.182	0.052	0.122	0.001
Unweighted unifrac full tree	0.247	0.005	0.101	0.001
Weighted normalized unifrac	0.334	0.002	0.164	0.001
Binary serensen dice	0.379	0.001	0.098	0.001
Binary chisq	0.229	0.001	0.061	0.001
Binary chord	0.386	0.001	0.067	0.001
Binary euclidean	0.431	0.001	0.066	0.001
Binary hamming	0.431	0.001	0.096	0.001
Binary jaccard	0.379	0.001	0.081	0.001
Binary lennon	0.454	0.001	0.107	0.001
Binary ochiai	0.386	0.001	0.098	0.001
Binary pearson	0.432	0.001	0.097	0.001

Table S10 Intergroup difference analysis between G2 and G4 using ANOSIM and Adonis test based on 29 different distance algorithms

Distance algorithms	ANOSIM test		Adonis test	
	R value	*P*	R^2 value	*P*
Bray curtis	0.879	0.001	0.286	0.001
Unweighted unifrac	0.651	0.001	0.179	0.001
Weighted unifrac	0.847	0.001	0.558	0.001
Euclidean	0.716	0.001	0.461	0.001
Abund jaccard	0.893	0.001	0.653	0.001
Bray curtis faith	0.879	0.001	0.286	0.001
Bray curtis magurran	0.879	0.001	0.286	0.001
Canberra	0.637	0.001	0.090	0.001
Chisq	0.206	0.001	0.106	0.001
Chord	0.957	0.001	0.365	0.001
Hellinger	0.908	0.001	0.274	0.001
Kulcynski	0.879	0.001	0.286	0.001
Manhattan	0.879	0.001	0.286	0.001
Morista horn	0.964	0.001	0.525	0.001
Person	0.954	0.001	0.544	0.001
Soergel	0.879	0.001	0.183	0.001
Spearman approx	0.865	0.001	0.252	0.001
Specprof	0.716	0.001	0.461	0.001
Unweighted unifrac full tree	0.768	0.001	0.236	0.001
Weighted normalized unifrac	0.912	0.001	0.555	0.001
Binary serensen dice	0.642	0.001	0.225	0.001
Binary chisq	0.487	0.001	0.101	0.001
Binary chord	0.644	0.001	0.126	0.001
Binary euclidean	0.883	0.001	0.135	0.001
Binary hamming	0.883	0.001	0.242	0.001
Binary jaccard	0.642	0.001	0.165	0.001
Binary lennon	0.534	0.001	0.183	0.001
Binary ochiai	0.644	0.001	0.220	0.001
Binary pearson	0.812	0.001	0.218	0.001

Table S11 Intergroup analysis of dominant phyla and genera in the gut microbiota of forest musk deer under different influencing factors. Significant P-values less than 0.05 are indicated in bold

Dominant bacteria	Probiotic groups		Seasonal groups		Age groups		
	G1-G3	G2-G4	G3-G5	G4-G6	G1-G2	G3-G4	G5-G6
Firmicutes	0.065	**0**	**0**	**0.004**	**0.001**	0.224	0.499
Bacteroidota	**0.011**	**0**	**0**	**0.005**	0.095	0.304	0.402
Proteobacteria	**0.001**	**0**	0.358	0.394	**0.001**	0.978	0.49
Actinobacteriota	**0.001**	**0.002**	**0.01**	0.636	0.113	0.130	0.25
Christensenellaceae R-7 group	0.235	**0**	**0**	**0**	**0**	0.449	0.787
UCG-005	0.071	**0.009**	**0**	0.607	**0.043**	**0.017**	0.13
Acinetobacter	**0.001**	**0**	**0.007**	0.198	**0.001**	0.223	0.534
Bacteroides	0.965	0.344	**0**	**0.005**	**0.025**	0.058	0.57
Rikenellaceae RC9 gut_group	0.692	**0.007**	0.304	**0.002**	**0.028**	0.839	0.185
Alistipes	0.826	**0.055**	0.123	0.213	**0.022**	0.137	0.626
NK4A214 group	**0.005**	0.685	0.079	**0.006**	0.159	0.213	0.797
Prevotellaceae UCG-004	0.428	**0**	0.534	0.304	0.071	0.394	0.665
Ruminococcus	0.965	**0**	0.051	0.204	**0.002**	0.978	0.449
Monoglobus	0.428	**0**	0.256	**0.001**	0.135	**0.03**	0.808

Table S12 Mantel Test analysis of intergroup differences in forest musk deer gut microbiota under different influencing factors using various distance algorithm. Statistically significant differences in effect sizes (*r*) are indicated in bold.

Distance algorithms	Probiotic groups		Seasonal groups		Age groups	
	r	*P*	*r*	*P*	*r*	*P*
Bray crutis	**0.596**	**0.001**	**0.207**	**0.001**	0.030	0.023
Abund jaccard	**0.693**	**0.001**	**0.149**	**0.001**	0.013	0.222
Bray chisq	**0.427**	**0.001**	**-0.035**	**0.351**	-0.010	0.413
Bray chord	**0.577**	**0.001**	**0.076**	**0.024**	0.005	0.666
Bray euclidean	**0.610**	**0.001**	**0.280**	**0.001**	**0.035**	**0.025**
Bray hamming	**0.606**	**0.001**	**0.272**	**0.001**	**0.032**	**0.020**
Bray jaccard	**0.576**	**0.001**	**0.077**	**0.018**	0.005	0.721
Bray lennon	**0.525**	**0.001**	**0.053**	**0.075**	0.008	0.455
Bray ochiai	**0.557**	**0.001**	**0.066**	**0.044**	0.004	0.740
Bray pearon dice	**0.604**	**0.001**	**0.183**	**0.001**	0.019	0.089
Bray crutis faith	**0.596**	**0.001**	**0.207**	**0.001**	**0.030**	**0.034**
Bray crutis magurran	**0.596**	**0.001**	**0.207**	**0.001**	**0.030**	**0.034**
Camberra	**0.566**	**0.001**	**0.138**	**0.001**	0.007	0.587
Chisq	**0.283**	**0.001**	-0.035	0.333	0.000	0.975
Chord	**0.545**	**0.001**	**0.243**	**0.001**	**0.047**	**0.005**
Euclidean	**0.547**	**0.001**	-0.026	0.464	-0.026	0.464
Gower	**-0.163**	**0.002**	**0.361**	**0.001**	**0.038**	**0.013**
Hellinger	**0.637**	**0.001**	**0.192**	**0.001**	**0.025**	**0.046**
Kulczyski	**0.596**	**0.001**	**0.207**	**0.001**	**0.030**	**0.033**
Manhattan	**0.596**	**0.001**	**0.207**	**0.001**	**0.030**	**0.034**
Morisita horn	**0.592**	**0.001**	**0.225**	**0.001**	**0.037**	**0.021**
Pearson	**0.579**	**0.001**	**0.238**	**0.001**	**0.037**	**0.022**
Soergel	**0.594**	**0.001**	**0.226**	**0.001**	**0.034**	**0.018**
Spearman approx	**0.603**	**0.001**	**0.308**	**0.001**	0.036	0.022
Specprof	**0.547**	**0.001**	-0.026	0.407	0.009	0.429
Unifrac	**0.549**	**0.001**	**0.104**	**0.003**	0.026	0.060
Unweighted unifrac	**0.549**	**0.001**	**0.104**	**0.002**	0.026	0.038
Unweighted normalized unifrac	**0.592**	**0.001**	**0.227**	**0.001**	0.015	0.172
Weighted unifrac	**0.612**	**0.001**	**0.125**	**0.002**	0.009	0.392

Table S13 Analysis of differences in gut microbiota in forest musk deer under varying influencing factors based on linear regression.

Diversity Type		Probiotic groups		Seasonal groups		Age groups	
		R^2	*P*	R^2	*P*	R^2	*P*
α diversity	Sobs	0.400	0	0.217	0	0.051	0.018
	Shannon	0.524	0	0.256	0	0.039	0.039
	Chao1	0.306	0	0.176	0	0.052	0.017
β diversity	PCA	0.582	0	0.134	0.0001	0.069	0.006
	PCoA	0.751	0	0.310	0	0.069	0.005
	NMDS	0.683	0	0.255	0	0.048	0.022

Fig. S1 Differentiation analysis of the assembly process under different influencing factors.

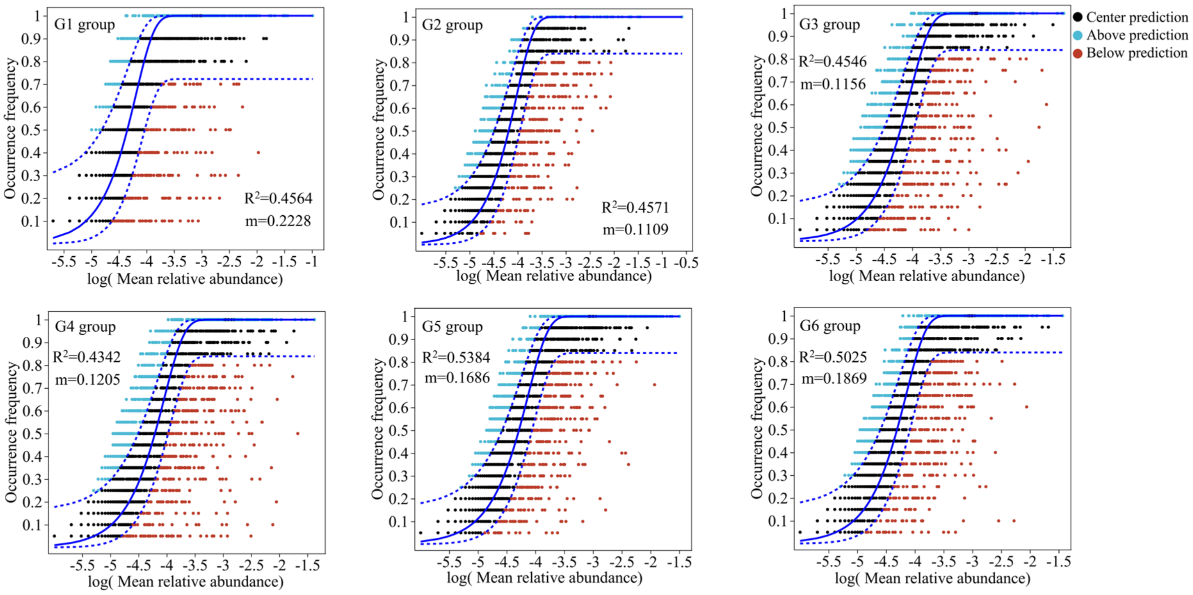

Supplement: Supplementary file 1 [file microorganisms-13-01991-s001.zip › microorganisms-3786831-supplementary.pdf]
